# Sex Differences in Alloimmunity and Other Immunological Considerations in Organ Transplantation

**DOI:** 10.1111/imr.70104

**Published:** 2026-03

**Authors:** Whitney A. Perry, Pritha Sen

**Affiliations:** 1 Division of Geographic Medicine and Infectious Diseases, Tufts Medical Center, Boston, Massachusetts, USA; 2 Transplant, Oncology, and Immunocompromised Host Group, Division of Infectious Diseases, Brigham and Women’s Hospital, Boston, Massachusetts, USA; 3 Dana-Farber Cancer Institute, Boston, Massachusetts, USA; 4 Hematology and Oncology Group, Division of Infectious Diseases, Massachusetts General Hospital, Boston, Massachusetts, USA; 5 Harvard Medical School, Boston, Massachusetts, USA

**Keywords:** aging, cell lineages and subsets, infectious diseases, major histocompatibility complex, molecules, processes, T cells, therapeutic approaches, transplantation, viral

## Abstract

Sex-based differences in human immunity are well-described in the general population, cumulatively favoring a more hyperin-flammatory immune response among females in different scenarios. It is imperative to understand how this applies in the setting of transplantation, where dynamic immune function determines key clinical outcomes, including graft rejection and responses to infection. Sex differences unique to transplant immunity begin at the chromosomal level and are influenced by steroid hormones and sex-specific factors ranging from pre-existing pregnancy-induced alloimmunization to differential metabolism of routine immunosuppressive medications. Given active efforts for greater sex and gender equity in transplantation, and with increasing transplantation of older candidates, the unique immunological considerations at the cross-roads of biological sex and age are also critical to consider. Age is an important modifier of the relationship between sex and immune-related outcomes as young females appear to have a greater risk for poor outcomes related to alloimmunity compared to similarly-aged male counterparts, particularly when receiving an organ from a male donor. However, this risk does not endure in post-menopausal age categories and there are some limited data to suggest, in fact, that post-menopausal women may have a greater risk for transplant-related infection. Greater efforts to incorporate sex as a biological variable into transplant research will lead to a more tailored strategy for immunosuppressive management, rejection surveillance, infection prophylaxis and other considerations for optimization of transplant outcomes.

## 1 | Introduction

A growing understanding of the impact of sex as a biological variable in solid organ transplantation has prompted a call to action to better incorporate sex into transplant research and transplant policy decisions [1–4]. Following organ transplantation, patients require lifelong immunosuppression to protect the organ graft from acute or chronic rejection. Successful outcomes are highly dependent on a nuanced understanding of the many factors that affect “the net state of immunosuppression;” this is a concept in transplant literature that acknowledges the layered, dynamic and inter-related contribution of donor characteristics, recipient characteristics, organ immunogenicity, immunosuppressive medications, and environment on a patient’s functional immunity [5]. Transplant clinicians must continuously evaluate an individual patient’s net state of immunosuppression and consider the competing demands of minimizing rejection, infection, medication toxicity, and other complications of excess immunosuppression.

Donor and recipient biological sex introduce important layers of immune complexity that indisputably affect transplant outcomes. Traditionally, women have been a minority of all organ transplant recipients, ranging between 25% and 40% depending on organ type and, accordingly, have been under-represented in transplant research [6]. Sex-based differences in immunity are well-described in the general population, cumulatively favoring a more robust immune response among females [7]. Understanding how this applies to the transplant setting, where net state of immunosuppression drives outcomes, is imperative. Isolating the effect of biological sex in transplantation is complicated by the inextricable effects of gender at various stages of the transplant process. Figure 1 illustrates a select list of sex- and gender-related factors in the pre-transplant, peri-transplant, and post-transplant periods that directly or indirectly affect transplant outcomes [8]. Additionally, and equally important variable that must be incorporated into any discussion of sex as a biological variable is age, which is an indisputable modifier of the relationship between sex and transplant outcomes. This review will primarily focus on biological sex as it impacts important transplant outcomes dependent on net state of immunosuppression, including cell- and antibody-mediated rejection, long-term graft survival, infection incidence, infection severity, and mortality.

To summarize the current understanding of the complex relationship between biological sex and immunity in the context of solid organ transplantation, we first highlight clinical and epidemiologic data that substantiate the relationship between sex, age, and graft outcomes. Further, we describe genetic, hormonal, and pregnancy-specific considerations that lead to sex differences in alloimmunity and infection risk.

## 2 | Clinical and Epidemiologic Data Supporting Sex as a Determinant of Organ Transplantation Outcomes

Although sex differences in transplant alloimmunity have been described in smaller studies for decades, several recent analyses utilizing large national registry databases have substantiated these conclusions and helped to parse out additional modifying factors. Because of the large number of variables that affect graft survival and because of the relatively smaller number of female organ recipients, single or limited multicenter studies are often not well-suited to show sex differences. However, databases like the Scientific Registry of Transplant Recipients (SRTR) in the United States and similar resources in other countries provide sufficient power, allowing for stratification by organ type, donor and recipient sex combinations, and age. These large analyses identify disparity in clinical outcomes (i.e., acute rejection, long-term graft failure) and provide a strong basis for more granular investigation into underlying mechanisms of observed differences.

Each organ type must be studied individually as each carries its own intrinsic characteristics such as underlying and comorbid disease states, organ-specific immunogenicity, protocol-directed immunosuppressive regimen, anti-rejection surveillance strategies, and infection prophylaxis and surveillance cadence. More minor or sporadic center-to-center variation in these domains can be overcome by large sample size. At present, there is no consideration of sex in the protocolled management of any organ group; male and female recipients typically receive the same immunosuppressive drugs at identical doses and follow the same rejection and infection surveillance strategy. The three most commonly-transplanted organs—kidney, liver, and heart—are discussed here; while other organ types likely have similar trends, large dedicated studies investigating sex differences in these subpopulations are lacking.

### 2.1 | Sex Differences in Kidney Transplant Outcomes

The majority of graft failure following kidney transplantation is due to dysregulated immunity [9]. Several key studies identify female recipients as having significantly greater rates of graft loss and highlight the modifying role of age and donor sex on graft failure. One of the most informative studies demonstrating the impact of sex on solid organ allograft failure evaluated nearly 160,000 kidney transplants occurring in the US in an 18-year period, stratified by age category (0–14, 15–24, 25–44, and > 45 years) and donor sex. In the setting of a male donor, female recipients in all age categories had an increased adjusted hazard of graft failure, though effect size decreased substantially in the oldest age category (0–14 years: adjusted hazard ratio [aHR] 1.51 [95% confidence interval [CI] 1.19 to 1.90]; 15–24 years: 1.37 [1.18 to 1.59]; 25–44 years: 1.14 [1.03 to 1.26]; 45 years: 1.05 [1.01 to 1.09]) [10]. In contrast, in the setting of a female donor, only female recipients age 15–24 years had a higher graft failure risk compared with male counterparts; in fact, the relationship reversed in the oldest age category (> 45 years) with a lower graft failure rate among female recipients compared with male recipients (aHR 0.95 [CI 0.91 to 0.99]) [10].

A subsequent study confirmed and expanded upon these findings, pooling US data with two other large non-US databases, for a total cohort of over 400,000 kidney transplants [11]. This study also provided an additional upper age category, separating 45–60 years from > 60 years which provided greater resolution for comparison, and concluded that previously-seen relationships among older donor-recipient sex combinations (less graft failure among females) were even more pronounced in the oldest category, regardless of donor sex. These findings are summarized in Table 1.

The effect modification by both donor sex and by recipient age are the foundation for hypotheses that recipient sex differences in graft outcomes are duly driven by both sex chromosome mismatch (i.e., female immune response to male-specific HY antigen) and by the broad-ranging effects of sex hormones on immune reactivity. Honing in on the instrumental role of post-menopausal hormonal shifts in older female transplant recipients, a third recent registry study confirms the statistically significant interaction between recipient sex and age on 5-year death-censored graft survival, regardless of donor sex [14]. Further, they compare the frequency of acute rejection among younger versus older female recipients, concluding that within 1 year of transplant, young female recipients (age 15–35 years) had twice the odds of an acute rejection event compared with older female recipients (age 55–75 years) [14]. Lower rates of acute rejection among older compared to younger females suggest a dampening of the predilection for alloimmune reactivity with post-menopausal hormonal shifts.

Importantly, the clinical outcome of excess mortality has been evaluated in similar analyses of kidney transplant registry cohorts. Indeed, as expected based on graft loss data, young female kidney recipients, particularly those who received an organ from a male donor, show higher relative excess risk of mortality than male recipients [15]. However, in older age groups (45–59 and > 60 years), where graft loss is lower than male counterparts, the excess mortality risk among females is more challenging to explain. This incongruence highlights the complexity of this patient population and hearkens back to the central concept of net state of immune suppression. For older women, causes of mortality outside of graft failure (i.e., infection, malignancy, or other adverse effects of immune suppression) may be contributing to this excess mortality risk [16].

### 2.2 Sex Differences in Heart Transplant Outcomes

Although organ types have different immunogenicities and other idiosyncrasies, the propensity for young female recipients to have poor outcomes related to alloimmunity is observed in the field of heart transplantation as well [12, 17–20].

A multicenter observational study on acute rejection within the first post-transplant year by active surveillance with endomyocardial biopsy demonstrated that women experience a greater incidence of acute rejection [17]. In this study, mild rejection was observed with high frequency among both sexes, but women had over twice the odds of moderate or severe grade rejection (14% among women versus 5%, odds ratio 2.62, *p* = 0.025) [17]. Accordingly, women also had greater odds of hospitalization for acute rejection (OR 2.35, *p* = 0.04) [17]. Importantly, this study did not stratify by age or donor sex; however, the mean age among females was six years younger than that of males. The tendency for earlier, greater cumulative incidence, and more severe episodes of acute rejection in females is supported by earlier literature as well [18, 21].

Graft rejection can occur in the form of cell-mediated rejection or antibody-mediated rejection (AMR). Females commonly have higher pre-formed antibody response to HLA antigens prior to transplant, often resulting from preceding pregnancy-induced alloimmunization. This is a risk factor for AMR. Women have a greater burden of AMR as measured by incidence (27 vs. 7%, *p* = 0.001) and histologic score at various time points up to 36 months post-transplantation [22]. There is also an association between AMR and onset of severe coronary allograft vasculopathy (CAV) (HR 7.0; 95% CI 1.5–31.5) [22], a poor long-term outcome of cardiac transplantation occurring in about 30% of heart transplant recipients by five years post-transplant [19]. Although women have a greater burden of AMR and AMR is associated with CAV, the pathogenesis of this disease is multifactorial and the overall incidence is actually greater in male recipients [19].

Several studies looking at recipient sex in heart transplant recipients consistently demonstrate lower long-term graft survival among pre-menopausal females compared to male counterparts and to post-menopausal females [12, 23]. When incorporating donor sex, comparing by age category, and adjusting for other confounding variables, significant differences are limited only to certain subgroups [12]. Table 1 summarizes the direction of these findings in comparison with other organ groups. In the setting of a male donor, female recipients in young and old age categories have greater rates of graft failure compared with male recipients; the largest effect is seen in young girls and women (0–12 years: HR 1.36 [CI 1.03–1.81]; 13–24 years: 1.43 [1.09–1.88]), and the smallest difference is seen in the oldest age category (≥ 45 years: 1.16 [1.06–1.27]) [12]. In the setting of a female donor, graft failure rates do not significantly differ by sex in any category, though trends in the youngest age categories favor higher failure rates in females [12]. Cause of graft failure is not included in this analysis. It is important to recognize that one major source of graft failure, CAV, which has both immune and non-immune related risk factors, is more common among men [19]. Mortality data pooled from two large registry databases mirror graft failure findings [24].

### 2.3 Sex Differences in Liver Transplant Outcomes

Compared with kidney or heart grafts, the liver graft is physiologically less immunogenic, allows for greater tolerance, and therefore requires less systemic immune suppression to prevent rejection. Lower immunogenicity and immunosuppression likely explain why comparison of liver graft failure by recipient sex, stratified by donor sex, shows no statistically significant difference in nearly any age group. Similar to previously described studies of sex effect on graft failure rates in heart and kidney groups, large registry cohorts allow for appropriate stratification by donor sex and recipient age. An analysis of over three decades of liver transplants in the SRTR database using multivariable adjusted Cox modeling, showed graft failure was overall the same between sexes [13]. The direction of the hazard estimates is, in some cases, the same as other organs, but the magnitude of these differences is diminished and largely fails to meet statistical significance (Table 1). Specifically, in the setting of a male donor, young females may have marginally higher risk of graft failure but these results are not statistically significant. The trend entirely disappears in the oldest age category (> 45 years) where graft outcomes appear equal between sexes. Interestingly, when the donor is female, the only significant difference is in women over 45 years who have lower graft failure rates than male counterparts (adjusted HR 0.85 (0.81, 0.89)).

Although population-level sex differences in liver graft survival are not observed (except in the single subgroup stated above), it is important to mention that liver transplantation has its own unique challenges leading to sex disparity in allocation practices related to size, frailty, and intrinsic disadvantages in the scoring system used to prioritize waitlist candidates [25]. This is not strictly related to sex differences in immunity but may have indirect downstream effects related to waitlist time, quality of donor matching, conditional survival, and other factors. It is also noteworthy because the liver transplant community has recognized this important sex-based disparity and made efforts in recent years to adjust the allocation system to improve equity and outcomes [26]. There is additional work to do to achieve true equity in liver transplantation but this action provides evidence that sex differences research can be used to drive meaningful improvement in the field of transplantation.

### 2.4 Sex Differences in Transplant-Related Infection

Due to the immunosuppressive medications required to minimize the risk of graft rejection, transplant-related infection is a major source of morbidity and mortality in organ transplantation. In the healthy non-transplant population, there is strong evidence of sex difference in host response to a range of pathogens, particularly viruses (i.e., hepatitis B, hepatitis C, SARS-CoV-2), and to immunization (i.e., influenza) [7, 27, 28]. Commonly, immune response to viral infection tends to be more robust in females as compared to males, leading to swifter viral clearance, and in some cases, a greater risk of immunopathology [29]. This is highly relevant in the transplant population where routine medications specifically target cell-mediated immunity and lead to impaired response to viruses such as cytomegalovirus (CMV), the most common and challenging post-transplant infection [30]. However, uniform dosing of these medications without consideration of differential drug metabolism by sex or age likely leads to excessive overall drug exposure in certain subpopulations such as older, post-menopausal women. Therefore, underlying immune biases favoring robust female response may be attenuated. Research on clinical outcomes in this area is more limited than that of sex differences in alloimmunity, but several smaller studies have begun to inform our understanding for the converse risk of transplant-related infection.

The lower rate of graft rejection and graft failure among women in post-menopausal age groups suggests that the net state of immunosuppression—resulting from intrinsic and iatrogenic factors—is higher in this group. Consistent with this, multiple studies demonstrate significantly lower peripheral blood lymphocyte counts and serum total immunoglobulin G among post-menopausal women compared with both pre-menopausal women and all men [31, 32]. One of these analyses also identified a higher cumulative incidence of infection in females > 50 years, though that aspect of the analysis had limitations [31]. Nonetheless, prior studies have repeatedly shown that low levels of both lymphocytes and immunoglobulins are independently associated with post-transplant CMV, non-CMV infection, and mortality [33–39].

Clinically, older female organ recipients are more likely to have CMV reactivation [40, 41] and those with CMV infection have over four times the odds of tissue-invasive disease compared with other age and sex categories [42]. CMV in transplant recipients leads to substantial morbidity, mortality, and healthcare resource utilization [43, 44]. Aside from direct morbidity, CMV is associated with immune aging [45, 46], CAV in cardiac transplantation [47–49] and diminished host response to other pathogens [50–53]. Given suspected sex difference in immune control of CMV among transplant populations, sex effect in these indirect CMV outcomes should also be studied.

Taken together, it is critical to recognize the role of age in modifying sex effect in graft outcomes. Where targeted augmentation of immunosuppression may be appropriate to improve outcomes in young female recipients, the same strategy carries potential for harm in post-menopausal females who may suffer from excess immune suppression. It has never been more important to make this distinction as 20 years of Organ Procurement and Transplantation Network data demonstrate a steady increase in the proportion of female cardiac transplant recipients who are over the age of 50 years (Figure 2).

## 3 Mechanisms for Sex Differences in Organ Transplant Immunity

### 3.1 Genetic Components of Sex Differences in Organ Transplant Immunity

Prior to focusing our discussion on transplant-immunity specific differences related to biological sex, it is essential to recognize the landscape of literature outlining chromsomally-mediated sex differences that modulate immune function in the general population. Human males and females differentially express over 1800 transcripts, including 7% encoded on sex chromosomes and the remaining on autosomes [7]. The X chromosome not only encodes many hallmark genes of human immunity (*TLR7*, *IL2*, *IRAK1, FOXP3* and *CD40L*) it is also uniquely subject to the phenomena of mosaicism and X inactivation escape in 15% of cells, which may provide benefit in certain aspects of immune constitution [7, 29]. Differentially expressed genes on autosomes may result from steroid receptor signaling regulated by sex hormone binding and epigenetic modifications. The cumulative effect of the many downstream pathways to these sex-based genetic differences is a bias for more robust, hyperinflammatory immune response to self and non-self in females.

The sex-encoded minor histocompatibility antigen H-Y plays an important role in transplant alloimmunity. H-Y antigens, a group of minor histocompatibility antigens (mHA) encoded on the Y chromosome, are immunogenic proteins that have been demonstrated to break tolerance and promote rejection phenotypes in the context of pregnancy and transplantation [54–57]. Murine and rat alloimmunity models using skin graft transplants first identified the role for H-Y antigen in breaking tolerance [58–61]. Early seminal studies demonstrated that female mice rejected skin transplants from inbred male counterparts, and this rejection phenotype could be abrogated by pre-emptively treating the female mice with an infusion of splenic cells from the same male [58–61]. With subsequent challenge to the same female with an isologous skin graft from the male mouse, tolerance to a sex-related antigen, subsequently determined to be the H-Y related mHA, was achieved [59].

The presence of HLA-restricted cytotoxic T lymphocytes (CTL) against mHA on the Y chromosome was also identified by tetramer analysis [62–64]. *SMCY*, a gene identified between two zinc-finger genes on the short arm of the Y chromosome was found to encode an H-Y epitope, identifying the genetic foundation for sex-encoded antigenic differences [65]. In addition to SMCY, T cell responses against other minor histocompatibility antigens encoded on the Y chromosome, including from the dead-box helicase family (DBY), related to ubiquitination (DFFRY), RPS4Y, TMSB4Y, and UTY have also been described [66–69].

mHAs are drivers of rejection in HLA-identical solid organ transplantation, and allogenic responses to the H-Y antigen that drive rejection phenotypes in the context of transplantation are both T and B cell-related. HLA-restricted cytotoxicity against the H-Y antigen was first described in renal transplantation when a 22-year-old female who had received a living donor kidney transplant from her HLA-identical brother rejected the allograft two weeks after transplantation necessitating allograft explant [70]. Using mixed lymphocyte culture and cold target inhibition testing, her rejection was subsequently determined to be the result of a self HLA-A2-restricted CTL against a male-specific mHA. Interestingly, this same female had a successful transplant with retained graft function after receiving a subsequent kidney from an HLA-matched female, providing further evidence of the H-Y antigen’s immunogenic potential for causing acute rejection in solid organ transplantation [70]. mHA recognizing CTL have also been found to infiltrate renal allografts [71]. HLA allelic variants of MHC class I A01, 02, 24, 33; B07, 08, 27, 60 and class II DQB1, DRB1, and DRB3 have all been implicated in allogeneic immune responses to H-Y mHA [57, 72, 73]. Additionally, in one of the first descriptions of humoral responses against H-Y mHA in renal transplantation, female recipients of male kidneys developed antibodies to H-Y more frequently than other donor-recipient sex-matched and mismatched combinations, a clinical development which correlated with acute rejection and plasmablast allograft infiltrates on biopsy with antibodies to H-Y antigens RPS4Y1 most commonly detected [74].

The results from these small, case-report level translational human immunology studies, identifying the important role of H-Y mAH in renal allograft rejection, have been supported by epidemiologic data from large retrospective cohort studies. Analyses of The Collaborative Transplant Study, a registry of over 195,000 deceased donor allograft recipients receiving renal transplants between 1985 and 2004 from 400 transplant centers in 45 countries, demonstrated that females who received kidneys from male donors, as compared to same-sex donor-recipient pairs and male recipients of a kidney from a female donor, had increased risk of graft failure in the first year post-transplantation and this risk was maintained 10 years out from transplantation. This study provided indirect evidence that immunogenic responses to sex antigens, and H-Y antigen in particular, mediate graft rejection and failure in solid organ transplantation [75].

Beyond abdominal organ transplantation, the role of H-Y mHA in promoting alloimmunity has also been well documented in heart and lung transplantation. Regression analyses of pediatric cardiac transplantation cases performed between 1985 and 1989 have demonstrated that sex-encoded minor antigen H-Y mismatch is a significant factor associated with rejection at 3- and 12 months post-transplant; in cases where female recipients were transplanted with male hearts, the number of episodes of cardiac allograft rejection was significantly higher as compared to males and/or females receiving hearts from female donors [76]. Additionally, an International Society for Heart and Lung Transplantation registry cohort of cardiac transplants between 1990 and 2008, including greater than 60,000 recipients, demonstrated that donor-recipient sex mismatch is associated with poor 10-year survival [77]. However, there was no conclusive evidence of donor sex driving differences in CAV between male and female recipients [78]. The role of male-specific antigen in alloimmunity in musculoskeletal transplantation has been studied in rat models and has not conclusively demonstrated impact specifically to limb rejection [79].

T and B cell-mediated responses to the H-Y mHA have also been demonstrated to track with the development of graft-versus-host disease (GVHD), the rejection equivalent in hematopoietic stem cell transplantation (HSCT) [64, 80]. As described in an early seminal case report, a female patient with secondary myelodysplastic syndrome after treatment for Hodgkin’s lymphoma who had received a T lymphocyte-depleted bone marrow transplant from her HLA-identical father developed recipient cell-mediated toxicity against the H-Y antigen; with the persistence of H-Y epitope-specific CTL, the patient ultimately developed both graft failure and inhibition of growth in hematopoietic progenitor cells during the course of her bone marrow transplantation [81]. In HSCT, where immunity post-transplant is reconstituted predominantly by donor with recipient tissue taking the effective role of allograft, GVHD has been described in female donor to male recipient transplantation as compared to male-to-male donors [68, 82, 83]. H-Y antibodies were first described in the context of sex-mismatched HSCT with a large number of male patients with stem cell grafts from female donors developing antibody responses to mHA from the Y chromosome dead-box helicase gene *DBY* [84]. In a case study of anti-H-Y responses in a male HSCT recipient from an HLA-identical sister who developed chronic GVHD, donor-specific CD4^+^ T cell responses against both DBY and DBX were noted, as were antibody responses against DBY, further demonstrating how coordinated T and B cell responses to an H-Y antigen after allogeneic transplant can modulate GVHD [80].

### 3.2 Hormonal Influence on Sex Differences in Organ Transplant Immunity

Sex hormones have broad-ranging effects on both innate and adaptive arms of immunity. Most data available on this topic are not specific to the transplant population but inform potential mechanisms of sex differences in immunity-related transplant outcomes. Through the presence of nuclear and membrane-bound receptors for estrogens, progesterone, and androgens [29], sex hormones drive differences in immune responses by influencing both development and function of many immune cells types [85]. Hormone receptors are typically bound in the cytoplasm by their ligand, triggering dimerization and translocation to the nucleus where the complex binds to hormone response elements or engages transcriptional activators or co-repressors to modulate target gene expression [86]. In addition, several important alternative, overlapping, complementary pathways have been elucidated (i.e., G-protein-coupled estrogen receptors) [86]. A complete discussion of the effects of sex hormones on various immune cell populations is outside the scope of this review which will focus only on select features of sex hormones in transplant immunity.

#### 3.2.1 Estrogen

The estrogen hormone estradiol (E2), which is found in highest concentrations in females during reproductive years, can have either activating or tolerogenic effect on immune cells depending on concentration of the hormone, relative concentration of receptor subtypes, presence of corepressor or coactivator proteins, and the cell type [29, 87]. Broadly, E2 signaling affects cell proliferation, production of growth factors and cytokines (i.e., interferons, interleukin-6), receptor signaling pathways (i.e., NF-κB, STAT), transcription factors, and coregulators (i.e., c-Myc, c-Fos) [88]. E2 concentrations vary over the lifespan of women with reduction in circulating E2 after menopause. The reduction in E2 concentration likely results in an overall decrease in immune-activating influence and is a purported mechanism for the attenuated alloimmune response observed among post-menopausal versus pre-menopausal transplant recipients.

The instrumental role of E2 in alloimmunity is demonstrated in murine skin and heart transplant models [14]. Skin graft survival is significantly shorter in young female mice compared to male and older female mice [14]. In contrast, young ovariectomized female mice show longer graft survival, similar to that of old ovariectomized, old naïve, and sham-controlled female mice [14]. These findings are robust even in the presence of immunosuppression and also in a vascularized heart transplant model [14, 89]. Further, ovariectomized mice have reduced numbers of CD4^+^ and CD8^+^ lymphocytes, with reduced pro-inflammatory Th1 and Th17 responses, and augmented Treg response [14]. CD4^+^ T cell subsets show compromised proliferative capacity associated with reduced IFNγ production and increased cell death rate [14]. These findings emphasize the critical influence of E2 on T lymphocyte frequency and activation, the central immune cell population active in transplant rejection and the primary target of transplant immunosuppression.

The influence of E2 may begin immediately at transplantation, where it is thought to provide a protective effect against ischemia-reperfusion injury which occurs at the time of restoration of blood flow to a newly transplanted organ. Clinical and animal studies suggest that females are relatively protected from this peri-operative injury. Ischemia recovery is associated with recipient sex and not with donor sex [90]. Ischemic and reperfusion damage trigger reactive oxygen species production, cellular apoptosis, and tubular injury [91]. Damaged tubular cells express various proinflammatory cytokines/chemokines including monocyte chemoattractant protein-1 (MCP-1), TNF-α, IL-1β, IL-6, and IL-8 [91]. Innate immunity cells including macrophages, neutrophils, and natural killer cells, create an inflammatory cascade. The adaptive immune response is engaged by tubular cell expression of toll-like receptors and complement leading to T cell activity [91]. The clinical manifestation of ischemia reperfusion injury is delayed graft function.

In a mouse model, ovariectomized female mice exposed to warm ischemic reperfusion injury showed delayed renal recovery relative to female control mice [90]. In female estrogen receptor α knockout mice exposed to renal ischemia injury, elevated blood urea nitrogen indicated significant increased vulnerability to injury compared with controls and led to the death of all knockout mice at four days, compared with the survival of all controls [90]. Further, estrogen pre-treated females showed significant improvement in post-injury renal function compared with controls, though the same benefit was not clear in estrogen pre-treated males [90]. In a separate experimental model, pre-treatment with selective estrogen receptor modulator, raloxifene, in female mice resulted in significantly lower immediate indicators of renal injury after renal ischemia and significantly lower fibrosis at 30 days [92]. The more precise molecular mechanism of estrogen effect on reperfusion injury is not well-characterized and needs additional study.

Overall, little is known about sex differences in innate immunity in the setting of transplant. However, one additional area of interest is the effect of E2 on monocytes as this is the site of CMV latency and where CMV-mediated attenuation of host response to fungal pathogens occurs [50]. Classically, E2 signals through nuclear estrogen receptors α and β (ERα and ERβ), though membrane-bound estrogen receptors are also important. The relative expression of these receptors varies by immune cell type. Within lymphocyte subtypes (CD4^+^ T cells, CD8^+^ T cells, B cells), relative expression does not vary by sex [93]. Interestingly, however, monocyte expression of ERα, which can mediate tolerogenic effects [94, 95], is significantly greater in males and post-menopausal females compared with pre-menopausal females. Thus, in the context of clinical studies which show greater frequency of CMV reactivation [40] and more common end organ damage [42] among post-menopausal female recipients compared with younger female or male recipients, the role of E2 at this level invites further investigation.

#### 3.2.2 Progesterone

As with the E2, progesterone (P4) production also varies through the female life course, fluctuates with the menstrual cycle, and surges during pregnancy; P4 remains at higher concentrations among females compared with males throughout. Similar to other sex hormones, P4 operates through multiple nuclear and membrane-bound receptor subtypes on immune cells that are specific to P4 ligand, but P4 may also signal through certain other steroid hormone receptors as well [7]. P4 has an overall immune inhibitory effect on both innate and adaptive immunity. A key pathway to its inhibitory effects is through suppression of NF-κB activation and downstream inflammatory cytokines [88, 96].

Nuclear and membrane P4 receptors are expressed on female human T cells [97]. In both murine models and humans, P4 reduces CD4^+^ and CD8^+^ proliferation and activation and can induce apoptosis [88]. Additionally, P4 is a central player in promoting tolerance to the semi-allogenic fetus during pregnancy where it promotes Treg activity [98].

#### 3.2.3 Androgens

Androgen receptors are also present on multiple immune cell populations including thymocytes, peripheral T cells, B cells, neutrophils, monocytes, macrophages and type 2 lymphoid cells [7]. Testosterone and dihydrotestosterone, the androgens responsible for male sex characteristics, circulate in greatest concentration among adult men, but testosterone also surges during fetal development and early in life influencing immune cell development. Androgens typically exert an immune inhibitory influence [29]. Androgens influence T cell development in the thymus [88]. Androgen receptor signaling in thymic epithelial cells upregulates the expression of autoimmune regulator gene that facilitates negative selection [88]. Males therefore develop enhanced immune tolerance. Knockout of the androgen receptor results in increased thymopoiesis due to reduced negative selection, more robust T cell receptor signaling, and greater frequencies of T cells in the periphery [88, 99]. Androgen deficiency in castrated mice enables greater stimulated T cell proliferation, and greater antigen- and tissue-specific activation compared to T cells from sham-treated controls [100].

Given the instrumental role of lymphocytes in alloimmune response and the male bias toward lower greater graft survival, especially in age categories where testosterone is expected to be at peak levels, investigators have searched for an association between testosterone levels and graft outcomes. One small study shows that low serum testosterone is associated with impaired cardiac graft function and low-grade rejection episodes in the early post-transplant period [101]. Conversely, however, rat models of kidney transplantation demonstrate a role for androgens in the development of chronic allograft nephropathy [102, 103]. Overall, there is very limited and inconclusive data on the role of androgens in alloimmunity; this remains an area for further investigation.

#### 3.2.4 Shifting Hormonal Milieu Following Transplant

One of the unique considerations in hormonal influence on immunity following organ transplantation is the shifting hormonal milieu that accompanies transplantation. Due to chronic end stage organ disease including end stage renal or heart disease, and liver cirrhosis, organ transplant recipients commonly enter transplant with substantial baseline hormone dysfunction. Underlying organ disease states and common associated comorbid conditions produce chronic inflammation, hypothalamic–pituitary axis dysfunction, altered metabolic pathways, and uremia [104]. These factors lead to hypogonadism in men and impaired ovulation in women.

Among men with heart failure, studies estimate the prevalence of low testosterone to be in the range of 25%–56% [105]. Men with end stage renal disease have the lowest testosterone levels compared with men with earlier stage chronic kidney disease and with male kidney transplant recipients [106]. Men with cirrhosis have low testosterone compounded by reduced clearance of estradiol, aromatization, and other metabolic disruptions [107].

Similarly, estrogen and progesterone levels are disrupted by hypothalamic–pituitary-ovarian dysfunction in pre-menopausal women with end stage organ disease [31, 32]. Up to half of women with end stage liver disease experience secondary amenorrhea and others experience oligomenorrhea or menometrorrhagia [108]. Among young women on dialysis, some studies document greater than 90% having menstrual disturbance [108, 109].

However, with improvement of underlying organ function following successful transplant, endocrine and metabolic pathways are restored and studies demonstrate that this allows for recovery of sex hormones to more typical levels for age and sex. Studies widely vary in estimated time to restoration of menses or normalization of serum steroid hormone levels, but the majority confirm some measure of these endpoints within the first 6–12 months [31, 107–110]. Alongside the recovery of estradiol to typical reproductive levels in young females in the first 6–12 months, the associated immune activating effects fit with a picture of greater alloimmunity as observed in clinical studies.

### 3.3 | Innate Immunity in Transplant

As with adaptive immunity, countless important sex-based differences in the innate immune pathways are well-described in the general population [7]. While these differences exist in the background population, there is a scarcity of research specifically related to sex-based differences in innate immunity in organ transplantation; this remains a critical area for further investigation.

### 3.4 | Adaptive Immunity in Transplant

Adaptive immunity is of central importance in organ transplantation due to the primary role of lymphocytes in alloantigen recognition and tissue rejection. Pharmacologic immunosuppression in transplantation primarily target pathways of lymphocyte activation and expansion. Conversely, excessive suppression of lymphocyte populations poses a serious risk for infection and other poor outcomes [33–35]. For this reason, an understanding of sex differences in T and B lymphocyte populations in the setting of transplantation is invaluable.

Female heart transplant recipients, on average, have a significantly higher total circulating lymphocyte count (absolute lymphocyte count, ALC) one month following transplant compared with males [39]. However, age is an important modifier of this relationship. Total circulating lymphocyte count at six months post-transplant among female heart and kidney recipients over age 50 years falls below a threshold repeatedly associated with CMV and other infections [31]. Further, in the study showing this finding, women in the older age category were post-menopausal and concomitantly demonstrated low mean estradiol level [31]. These post-menopausal women had significantly lower ALC compared with premenopausal women and all men [31]. Distribution of CD4^+^ subpopulations and serum interferon gamma levels showed similar trends.

#### 3.4.1 | T Lymphocytes

While this high-level clinical study on ALC in different age and sex strata is small, the results remain consistent both with previously-discussed large epidemiologic data showing lower graft survival in young females and greater graft survival in older females [11, 12] and with mouse transplant models showing the effect of estradiol on T cell populations [14]. Murine transplant models show not only a reduced number of post-transplant circulating CD4^+^ and CD8^+^ T cells in ovariectomized mice compared with sham controls but also show important differences in T cell function [14]. Circulating cells in the ovariectomized group show dampened Th1 (CD4^+^IFNγ^+^) and Th17 (CD4^+^IL-17^+^) responses and increased CD4^+^CD25^+^FOXP3^+^ Treg cells [14].

The inverse correlation of E2 with Treg cells is also supported by human data in young kidney transplant recipients [111]. In this group, FOXP3-expressing T cells show a lower frequency of Helios-expression, a marker of active Treg capacity, among females compared with males [111]. The difference was greatest among recipients over the age of 12 years [111].

#### 3.4.2 | B Lymphocytes and Humoral Immunity

In the general population, B cell frequency and antibody production are higher among females compared with males [29]. Humoral immunity creates a major source of sex disparity in transplantation. Panel reactive antibody assessment is a measure of the pre-existing humoral sensitization in a transplant candidate. This measure quantifies the percentage of MHC class I and II antigens commonly expressed on donor allograft cells for which the candidate has anti-human leukocyte antigen (HLA) antibodies. Calculated PRA (cPRA) levels are monitored routinely prior to transplant. Increased sensitization, even at low levels, is a risk factor for more frequent and severe rejection, CAV in cardiac transplantation, and death [20]. Pregnancy is the most common source of alloimmunization. Other contributing factors include use of a ventricular assist device, blood transfusion, and prior transplant. Due to the contribution of pregnancy, cPRA levels are higher in women [20]. Over 50% of women have positive PRA, and frequency increases with increasing parity (74% in women with greater than two deliveries) [112]. Females comprise 63% of the subset of transplant candidates characterized as very highly sensitized (> 98% cPRA) [113]. Sensitization has several negative impacts on transplant. First, it limits compatible donors and creates extended time on the transplant waitlist in search of a suitable match [114, 115]. Furthermore, living kidney donor options may be more restricted since spouse and offspring, frequently considered in the donor pool, may be incompatible due to direct alloimmunization from the candidate’s prior pregnancy [114]. Even after an acceptable donor is identified and transplant proceeds, memory B cells from pregnancy alloimmunization may rapidly produce antibody, purportedly resulting from either pre-existing memory T cells or plasma cells, leading to antibody-mediated rejection [115]. One small study found that in female heart transplant recipients, peak pre-transplant PRA level is associated with increased graft failure; whereas in male heart transplant recipients, peak PRA level is associated with CAV [20].

### 3.5 | Pregnancy and Alloimmunity

The concept of tolerance in pregnancy, whereby the maternal immune system does not attack or reject the fetus as it would an alloantigen, is both a conceptual and true immunological correlate to tolerance in solid organ transplantation. There are several hallmarks of how pregnancy as a model informs our understanding of solid organ transplantation including: dissemination of alloantigen; eliciting the development of anti-HLA antibodies; favoring Treg proliferation; and re-programming the cell states of fetal-specific maternal T cells [115–118].

Long-lived fetal “antigen pools,” initially distributed during pregnancy but then persistent in maternal circulation and tissue post-partum, can create a state of micro-chimerism and is associated with fetal-specific Tregs and upregulation of markers of exhaustion in CD8^+^ T cells, all of which enable fetal tolerance [119, 120]. Murine models of pregnancy have shown that while fetal-specific CD8^+^ T cells can be primed during pregnancy, they do not transform into pan-effector, cytokine-responsive or cytotoxic phenotypes [121, 122]. Instead, they maintain markers of exhaustion to remain in a state of T cell dysfunction that is situationally functional in that it “re-strain[s] maternal anti-fetal alloreactivity to promote reproductive fitness” [121–123].

Breaks in the tolerance state of pregnancy have been implicated in poor maternal-fetal outcomes and have implications for our understanding of sex-based mechanisms for graft rejection in transplantation. For example, females with a history of birth of male children are sensitized to the male fetus and develop H-Y alloimmunity, which in turn can increase risk of miscarriage during subsequent pregnancy; H-Y mHA alloimmunity has also been implicated as a potential mechanism for recurrent miscarriage after successful birth and both anti-H-Y antibodies and presence of H-Y restricted alleles have been associated with spontaneous rupture of membranes [124, 125]. Pregnancy is not only the most common alloimmunization event in human immunity, but each pregnancy is a unique and individual event that, genetically, has the potential to impact transplantation in females through the development of anti-HLA antibodies. Women listed for transplant are more likely both to have cPRA, and to have higher cPRA [113]. Several studies have also demonstrated that the risk of graft loss increases with gravidity [126].

### 3.6 | Metabolism and Tolerability of Immunosuppressive Drugs

The net state of immunosuppression [5] in organ transplant patients is highly influenced by iatrogenic immunosuppression. To protect the graft from acute and chronic rejection pathogenesis, transplant recipients receive lifelong immunosuppression beginning at the time of transplant. Because of the central role of lymphocytes in alloimmunity, anti-rejection medications primarily target cell-mediated immunity. Allowing for some variation by organ type and center, organ transplant recipients typically receive an induction phase followed by a maintenance phase of immunosuppression. Centers typically rely on protocol-driven management of these medications. Immunosuppressive medications are dosed at the highest levels in the first post-transplant year, followed by protocol-guided reduction to a stable long-term dose. The backbone of most contemporary immunosuppressive transplant regimens relies on some combination of three main drug classes including glucocorticoids (i.e., methylprednisone, prednisone), an antimetabolite (i.e., mycophenolate, azathioprine), and a calcineurin inhibitor (i.e., tacrolimus, cyclosporine). These protocols largely do not account for recipient sex.

#### 3.6.1 | Calcineurin Inhibitors

Calcineurin inhibitors act by blocking transcription of interleukin-2 and other cytokines within T lymphocytes, thereby limiting activation, proliferation, and differentiation of T cells. Tacrolimus, the most commonly used first-line calcineurin inhibitor in contemporary transplant medicine, has large intra- and interpatient pharmacokinetic variability and a narrow therapeutic window. Interpatient variability based on sex has been reported [127]. Female kidney transplant recipients appear to have greater clearance of tacrolimus [127] and cyclosporine [128], though additional investigation in patients with systemic lupus erythematosus actually demonstrates that the concentration of tacrolimus is significantly higher in women over 50 years than in women aged under 50 years [129]. This information is important for females in both age categories and further supports clinical data suggesting greater risk of alloimmune-driven outcomes in young females [14], contrasted by a greater net state of immunosuppression in older females [31].

#### 3.6.2 | Glucocorticoids

Glucocorticoids are used commonly in induction therapy and tapered at different rates according to organ transplant type and often varying by center and patient-specific factors. The typical duration varies from days to lifelong. Glucocorticoid reinitiation or augmentation is also a mainstay of treatment for rejection episodes. Studies of prednisolone in healthy volunteers, kidney, heart, and lung transplant recipients show that female sex and use of oral contraceptives are associated with lower clearance and greater dose-normalized exposure [130]. Studies were conflicting on whether clearance was reduced among transplant recipients over age 60 [130], though at least one nontransplant study shows unbound clearance of prednisolone is lower among postmenopausal females compared with pre-menopausal counterparts [131, 132].

#### 3.6.3 | Antimetabolites

Mycophenolate derivatives are first-line antimetabolite medications used in contemporary transplant medicine. Mycophenolate blocks the enzymatic production of *de novo* guanosine nucleotide synthesis, halting DNA and RNA synthesis in rapidly-dividing lymphocytes. Multiple studies demonstrate that male kidney transplant patients have higher mycophenolate clearance than female recipients [133, 134]. This is hypothesized to be due to E2 competition of binding sites that slow the glucuronidation and clearance of mycophenolate [134, 135].

#### 3.6.4 | Sex Differences in Tolerability of Immunosuppressive Medications

Medication toxicity is a substantial clinical burden in transplant patients who are unavoidably on a long list of routine post-transplant medications (2–3 immunosuppressives, 2–3 antimicrobial prophylaxis medications and myriad other disease-specific medications) in the immediate post-transplant period. Gender likely affects the likelihood of reporting and/or provider perception of medication-related symptoms, but metabolism and other features impacted by biological sex undoubtedly contribute to transplant-medication related complications [136], frequently leading to medication reduction or discontinuation, which ultimately may affect rates of rejection and graft failure [137].

## 4 | Conclusion and Future Direction

Disparities in both access to organs and transplantation outcomes (alloimmune syndromes, infection, graft failure, mortality) between female and male transplant recipients underscores the need to include, and rigorously evaluate, sex as a biological variable in transplantation [1, 2, 4]. The effects of sex on immunity are multifactorial, spanning mechanisms that are chromosomally-mediated (i.e., minor histocompatibility H-Y antigen), additionally influenced by dynamic sex hormone levels (i.e., E2, P4, and androgens) and further modified by sex-specific factors including pregnancy-driven alloimmunization and differential metabolism of routine immunosuppressive medications (i.e., slower clearance of mycophenolate). Sex-based differences in absolute lymphocyte counts and downstream T and B lymphocyte effector functions have been implicated in modulating transplant-specific immunity. Far less is known about sex differences in innate immunity as it relates to transplantation and is thus one central area that requires further investigation to meet the call to action for a better understanding of sex as a biological variable in transplantation. Additionally, with advances in transplant medicine that have enabled the consideration of older, higher risk candidates, the frequency of transplantation in candidates over age 50 is increasing [138, 139]. Given shifting hormonal milieus over time, defining the impact of age as a modifier of sex-related immunity outcomes in transplantation is critical. Tailored strategies to optimize transplantation outcomes, which may include targeted adjustment of protocol-driven immunosuppression, must account for the multiplicative effects of sex and age together to best meet the individual and unique needs of male and female transplant recipients across life course.

## Figures and Tables

**FIGURE 1 |:**
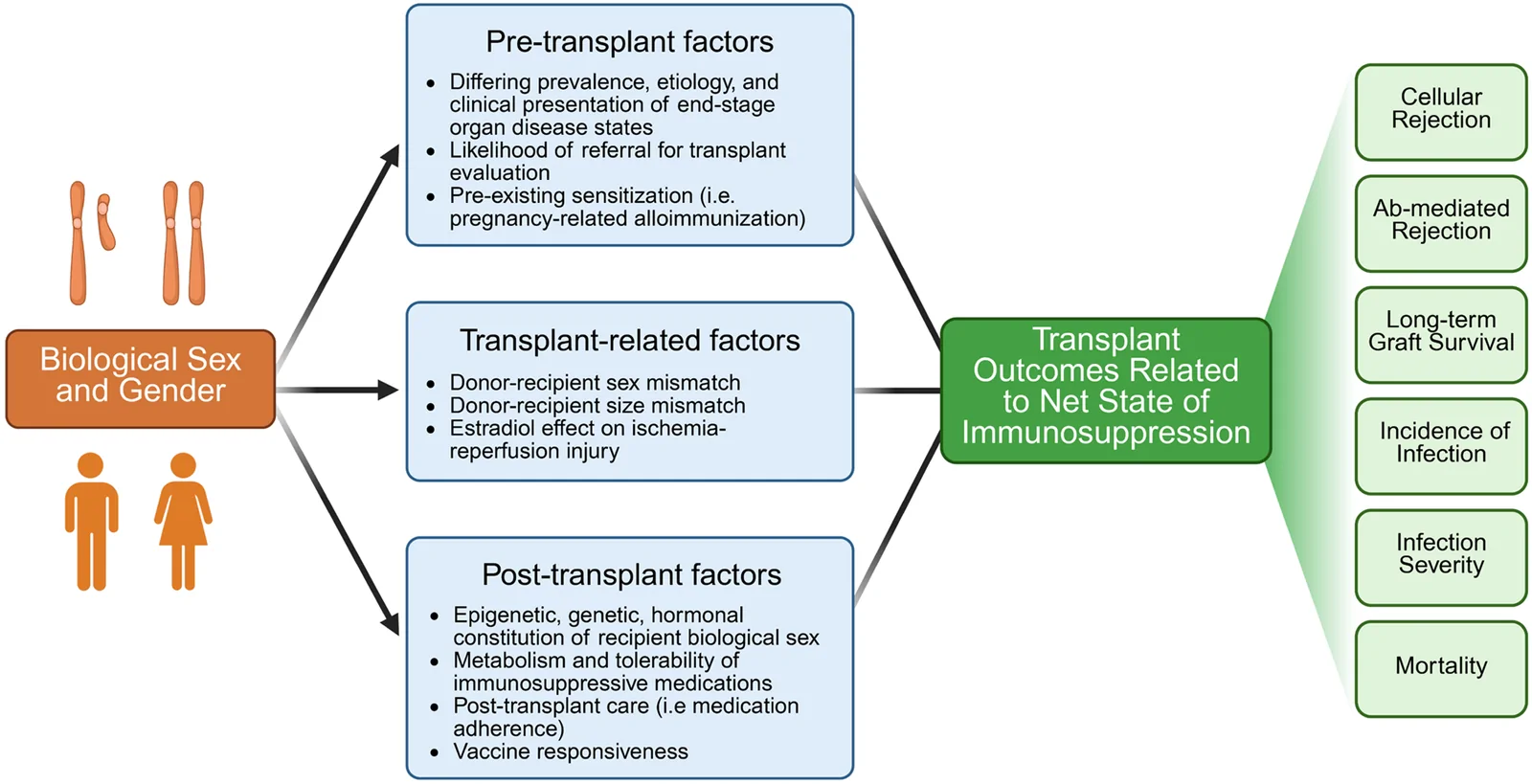
The impact of sex and gender on immunity-associated outcomes in organ transplantation. The combined influence of biological sex and gender are critical at pre-transplant, peri-transplant and post-transplant stages of clinical care. Sex is determined by biological characteristics, including sex chromosome composition, reproductive tissues and sex hormone concentrations. Gender refers to the socially constructed norms related to roles, behaviors, relationships. Sex and gender, while reciprocally intertwined, are separate entities, each with distinct impact on transplant outcomes at sequential stages. As an example, gender may influence circumstances of a patient's presentation to care (timing in disease course, communication of severity of symptoms, social support) and likelihood of referral for transplant evaluation. Pregnancy-related alloimmunization, a characteristic specific to biological female sex, may significantly limit potential donors and result in longer waitlist time or acceptance of a less optimally-matched organ.

**FIGURE 2 |:**
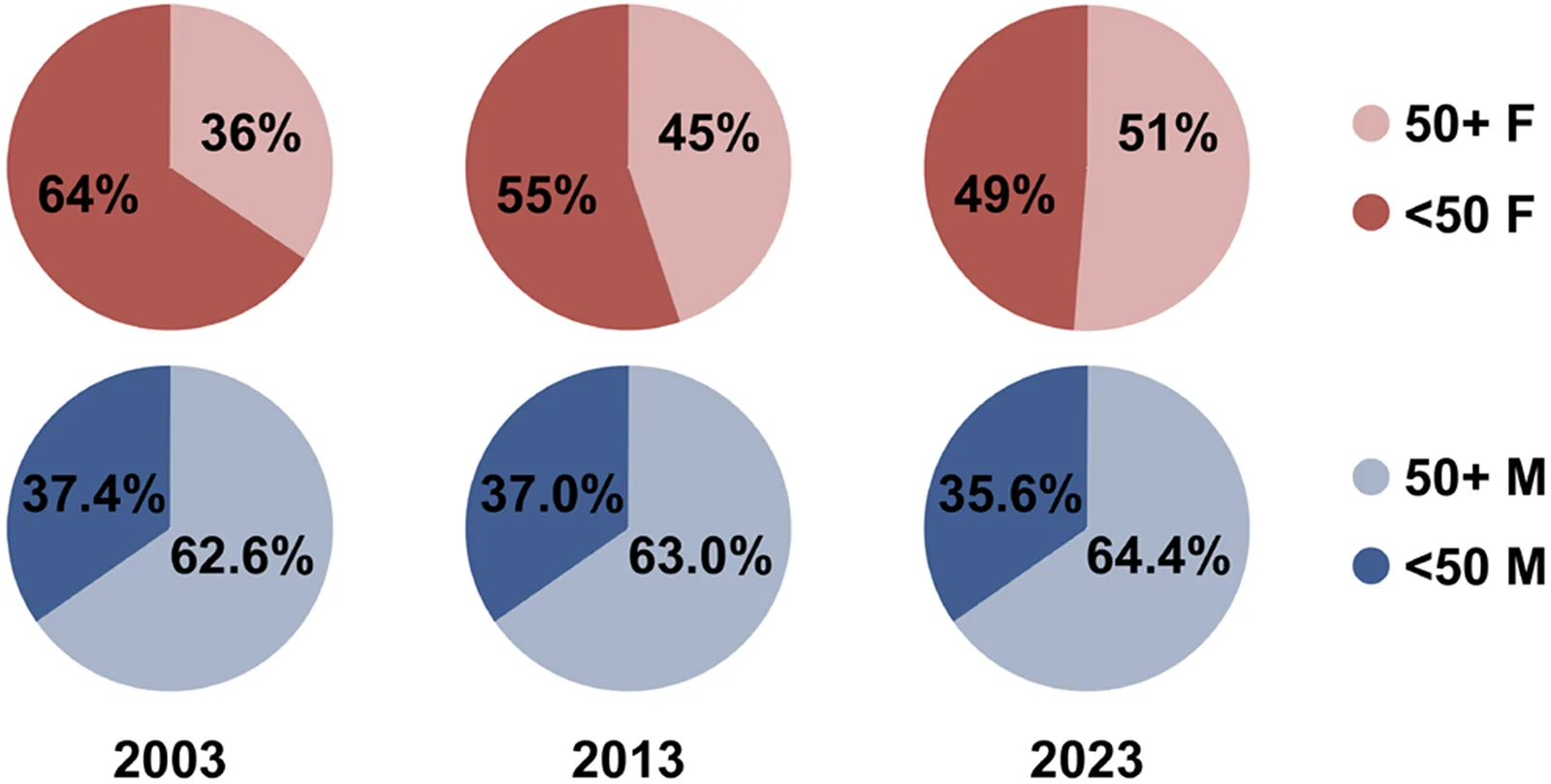
The proportion of female heart transplant recipients over the age of 50 has risen over a 20-year period while the distribution among male heart transplant recipients has remained stable. Based on Organ Procurement and Transplantation Network (OPTN) data as of Aug 5, 2024.

**TABLE 1 |:** Risk of graft failure represented as female versus to male organ recipient, stratified by age, donor sex, and transplanted organ.

	Heart^a^		Kidney^b^		Liver^c^	
Donor sex	Male	Female	Male	Female	Male	Female
Recipient age 0–12 years	↑	↑	↑	↑	↑	↓
Recipient age 13–34 years	↑	↑	↑	↑	↑	—
Recipient age 25–44 years	↑	—	↑	—	↑	↓
Recipient age 45–60 years	↑	—	—	↓	—	↓
Recipient age > 60 years			↓	↓		

*Note:* Direction of estimate indicated by arrow. Statistical significance indicated by shading; orange indicates statistically significant increased risk for females; blue indicates statistically significant decreased risk for females.

a Data summarized from Foster et al. [12].

b Representative data summarized from Vinson et al. [11].

c Data summarized from De Simone et al. [13].

## Data Availability

Data sharing not applicable—no new data generated.
